# Dietary Supplementation of Tannin-Extracts to Lambs: Effects on Meat Fatty Acids Composition and Stability and on Microbial Characteristics

**DOI:** 10.3390/foods8100469

**Published:** 2019-10-10

**Authors:** Luisa Biondi, Cinzia L. Randazzo, Nunziatina Russo, Alessandra Pino, Antonio Natalello, Koenraad Van Hoorde, Cinzia Caggia

**Affiliations:** 1 Department of Agriculture, Food and Environment, University of Catania, 95123 Catania, Italy; lubiondi@unict.it (L.B.); nunziatinarusso83@gmail.com (N.R.); alessandra.pino@unict.it (A.P.); antonio.natalello@unict.it (A.N.); ccaggia@unict.it (C.C.); 2 Service Foodborne Pathogens, Sciensano, B-1050 Brussels, Belgium; Koenraad.VanHoorde@sciensano.be; 3 Faculty of Bioscience Engineering, Department of Biotechnology, Laboratory of Brewing Science and Technology, Ghent University, B-9000 Ghent, Belgium

**Keywords:** hydrolysable tannins, condensed tannins, meat chemical composition, meat spoilage, *Pseudomonas* spp., meat shelf-life

## Abstract

Two extracts derived from plant material rich in hydrolysable (Tara, T; *Caesalpinia spinosa*) or condensed (Mimosa, M; *Acacia mearnsii*) tannins were added to lamb’s diet and their effects on meat quality and on microbial population were evaluated; a diet without tannins represented the Control (C). Meat pH, vitamin E, intramuscular fat content and muscle fatty acid composition were determined. Oxidative stability and microbiological analyses were performed on meat samples after 0, 4 and 7 days of refrigerated storage. Psychrotrophic bacteria were identified through MALDI-TOF MS analysis. Regarding meat fatty acids, Tara treatment decreased the percentage of monounsaturated fatty acids. The counts of all microbial groups were similar among dietary treatments at day 0, while a significant reduction of microbial loads was observed in T-group at day 7. *Pseudomonas fluorescens* group count was significantly affected by T extract supplementation. The MALDI-TOF MS identification revealed the dominance of *Pseudomonas fragi* species in all samples while *Pseudomonas lundensis*, *Brochothrix thermosphacta* and *Candida famata* were revealed only in control ones. In conclusions, the tannin extract supplementation is a promising dietary strategy to preserve lamb meat quality.

## 1. Introduction

In the last decade, the demand for natural preservative agents has increased due to the growing concern, among consumers, about the potential toxic effect of the synthetic antioxidants [1]. A number of reviews deal with the addition to feed or meat and meat products of plant extracts as natural antioxidants and antimicrobial agents [1,2,3]. According to Papuc et al. [3], commercially available polyphenols can extend meat shelf-life not only by improving its oxidative stability, but also by inhibiting bacterial growth. The most studied plant bioactive compounds in livestock feeding are phenolic compounds, such as tannins, and essential oils [4]. Animal responses to dietary tannins have been extensively reviewed mainly focusing on animal nutrition and production [5,6]. In the rumen, tannins can impair dietary fatty acids (FA) biohydrogenation (BH) influencing the FA profile of rumen content [7,8]. Thus, tannins could be exploited to favorably modulate FA composition of ruminant products [4,9]. However, controversial results on the effect of tannins on the meat FA composition have been observed in vivo [10,11]. Such inconsistency could be due to the type of tannin (e.g., hydrolysable or condensed), which can be differently metabolized in the rumen and can differently interact with feeds, bacteria and microbial metabolites [12]. It is well known that tannins are able to exert astringent, antiviral, antibacterial and antioxidant effects. The antimicrobial activity of tannins as well as their toxicity to bacteria, fungi and yeasts have long been recognized and several mechanisms, including inhibition of extracellular microbial enzymes, inhibition of oxidative phosphorylation or metal ions deprivation are involved [13]. It was already established that the use of plants rich in secondary compounds or the supplementation of plant-extract rich in polyphenols could represent a promising strategy for improving the meat oxidative stability, extending product shelf-life [4]. Therefore, the aim of the present study was to investigate the dietary supplementation of a source of hydrolysable (Tara; *Caesalpinia spinosa*) or condensed (Mimosa; *Acacia mearnsii*) tannins on oxidative stability and microbial population of lamb meat during its shelf-life.

## 2. Materials and Methods

### 2.1. Animals, Diets and Samplings Procedures

The applied procedures were in compliance with the European guidelines for the care and use of animals in research (Directive 2010/63/EU). The animals were handled by trained personnel. Fifteen male Sarda × Comisana lambs (body weight 19.6 kg ± 1.6) were selected at the age of 2 months and individually penned indoor in the experimental farm of the University of Catania. Lambs were randomly assigned to 3 dietary treatments (*n* = 5). The Control group (C) received a conventional concentrate containing (as fed): barley (480 g/kg), wheat bran (230 g/kg), dehydrated alfalfa hay (150 g/kg), soybean meal (100 g/kg), molasses (20 g/kg) and mineral-vitamin premix (20 g/kg). The other two treatments received the same basal diet of control in which 4% (as fed) of Mimosa (M group) or Tara (T group) tannin extracts was added. Tannins from Mimosa (*Acacia mearnsii*) and Tara (*Cesalpinia spinosa*) plants were extracted by maceration in water. The two extracts (commercial name: Mimosa OP^®^ and Tannino T80^®^, respectively for Mimosa and Tara) were purchased from Silvateam S.p.A. (San Michele Mondovì, Cuneo, Italy). All the diets were supplied in the form of a pellet and the tannin extracts were added to the diet ingredients before pelleting at the temperature of 40 °C. According the procedure described by Natalello et al. [14], total tannin concentration of experimental diets was 1.50, 22.3 and 25.3 g/kg dry matter (tannic acid equivalents). The chemical composition of basal diet is shown in Table 1. After a 9-day-adaptation period, consisting in a gradual introduction of the experimental diet, the lambs received their respective diet ad libitum; fresh water was always available. Individual feed intake and body weight were recorded during the experimental trial. At the end of feeding trial (75 days), all animals were slaughtered on the same day at a commercial abattoir according to the European Union welfare guidelines. After 24 h of storage at 4 °C, carcasses were halved and the entire longissimus thoracis et lumborum (LTL) muscles were removed from the right side, packed under vacuum and stored at −80 °C until tocopherol and fatty acids analysis. The LTL muscle from the left half-carcass was used to measure pH values by a pH meter (HI-110; Hanna Instruments) and then vacuum packaged and aged at +4 °C for 3 days. After that, 2 cm-thickness slices of LTL were prepared (one for each analysis and for each storage time) and stored at 4 °C for 0, 4 and 7 days, pending oxidative stability measurements and microbiological determination.

### 2.2. Sampling and Analyses of Feeds

Samples of experimental diets were collected weekly and immediately stored vacuum-packed at −20 °C. At the end of feeding trial, the weekly-collected feed samples were combined together in equal amount and the representative sample was analyzed for dry matter (DM), crude protein, ether extract, ash and fiber fractions (i.e., neutral and acid detergent fiber and acid detergent lignin) as described by Biondi et al. [15]. Furthermore, fatty acids and tocopherols of feedstuffs were extracted and quantified according the procedures reported in detail by Valenti et al. [16].

### 2.3. Vitamin E, Intramuscular Fat (IMF) and Fatty Acid Profile of Meat

The concentration of α-, γ- and δ-tocopherols (i.e., vitamin E) from muscle was determined as described by Luciano et al. [17]. In short, 2 g of muscle were homogenized in an ethanolic KOH solution (60%), containing BHT (0.06%), and incubated at 70 °C for 30 min. Tocopherols were extracted with hexane/ethyl acetate solution. Then, the extracts were dried under nitrogen, resuspended in acetonitrile and injected in a HPLC. The instrument information and chromatograph conditions were reported in [17]. The lipid concentration and fatty acid profile of meat were analysis as described by Natalello et al. [14]. In brief, intramuscular fat (IMF) was extracted from 5 g of muscle using chloroform/methanol (2:1, *v/v*). Then, lipid extract was methylated by a base-catalyzed procedure and the obtained FA methyl esters (FAME) were injected into a gas chromatograph as reported in [14]. Nonadecanoic acid (C19:0) was used as internal standard and FAs were expressed as g/100 g of total methylated fatty acids.

### 2.4. Meat Oxidative Stability Measurements

Meat oxidative stability over aerobic refrigerated storage was assessed on three slices (2 cm thickness) from the left LTL placed in polystyrene trays, covered with PVC film and stored at +4 °C. Each slice was used for measuring lipid oxidation and color stability at days 0 (after 2 h of blooming), 4 and 7. Lipid oxidation was measured as thiobarbituric acid reactive substances (TBARS) values according to the procedure described in Luciano et al. [18]. Meat color parameters were measured using a portable spectrophotometer (Minolta CM-2022, Tokyo, Japan), which recorded the following descriptors: lightness (L*), redness (a*), yellowness (b*), Chroma (C*) and Hue angle (H*), as well as the reflectance (R) spectra from 400 to 700 nm. The accumulation of metmyoglobin (MetMb) on the meat surface during storage was monitored by the ratio (K/S)_572_ ÷ (K/S)_525_ as described in [18]. This ratio decreases with increasing proportion of MetMb.

### 2.5. Microbiological Analysis

As for oxidative stability measurement, the microbiological analysis was evaluated at 0, 4 and 7 days of storage at 4 °C. At each sampling time, 25 g of sample, aseptically weighed, was transferred into a stomacher bag and homogenized with peptone water 0.1% *w*/*w* (Oxoid) for 2–3 min. Ten-fold dilutions were made and plated in triplicate on the following agar media and conditions: Plate Count Agar (PCA) incubated at 32 ± 2 °C for 48 h and at 4 °C for 7 days, for total mesophilic bacteria and total psychrotrophics, respectively; Pseudomonas Agar, supplemented with Cetrimide, Fucidine and Cephaloridine, incubated at 30 °C for 48 h for *Pseudomonas* spp. detection; Violet Red Bile Glucose agar, aerobically incubated at 37 °C for 24 h, for *Enterobacteriaceae* count; Brilliance Salmonella agar, supplemented with Salmonella selective supplement, incubated at 37 °C for 24 ± 3h, was used for *Salmonella* spp. count according to ISO 6579:2002 + A1:2007; Chromogenic *E. coli* incubated at 37 °C for 24 h, for *Escherichia coli* determination; Campylobacter Selective Agar (Preston) was used for *Campylobacter* spp. detection using the selective enrichment broth technique. All media were purchased at Oxoid, Milan, Italy. Results were expressed as log_10_ CFU/mL.

### 2.6. Isolation and Genetic Identification of Psychrotrophic Bacteria

From each PCA plate of T, M and C samples, previously incubated at 4 °C for 7 days, at 0, 4, and 7 days of refrigerated storage, 20% of the total number of colonies were randomly selected, purified, checked for catalase activity and Gram reaction, and microscopically examined before storing at −80 °C in liquid culture using 20% glycerol. Total genomic DNA of isolates was extracted from overnight cultures according to the method described by Pino et al. [19]. DNA concentration and quality were assessed by measuring the optical density at 260 nm using Fluorometer Qubit (Invitrogen, Carlsbad, CA, USA). All isolates were clustered by PCR-RFLP analysis, using primer pairs, PCR conditions and restriction endonucleases reported by Franzetti and Scarpellini [20]. The isolates of each PCR-RFLP cluster were subsequently subjected to species identification by MALDI-TOF MS analysis according to Russo et al. [21].

### 2.7. Statistical Analysis

The effect of the dietary treatment on muscle pH, intramuscular fat and fatty acids was assessed by means of one-way analysis of variance (ANOVA). Each animal represented an experimental unit. The oxidative stability (color descriptors, metmyoglobin and TBARS) and the microbiological (mesophilic and psychrotrophic bacteria, *E. coli*, *Enterobacteriaceae* and *Pseudomonas* spp.) data in meat were analyzed using a mixed model procedure for repeated measures. The fixed factors in the model were the dietary treatment (C, T and M), the time of storage (Time: days 0, 4, 7) and their interaction (Diet × Time), while the individual animal was included as a random factor. Differences between means were assessed using the Tukey’s adjustment for multiple comparisons. Effects and differences were declared significant when *p* ≤ 0.05, while trends toward significance where considered when 0.05 < *p* ≤ 0.1. Statistical analyses were performed with the statistical software Minitab, version 16 (Minitab Inc., State College, PA, USA).

## 3. Results

### 3.1. Animal Performance Parameters

Dietary treatments influenced the dry matter intake (*p* = 0.031), with lower intake for T-fed lambs (1.05 kg/day per lamb) as compared to M group (1.22 kg/day per lamb), while no difference was observed among Control (1.21 kg/day per lamb) and tannin groups (*p* > 0.05). Meanwhile, the other performances parameters were not affected by the experimental diets. Indeed, final body weight (average 35.3 ± 2.70 kg; *p* = 0.658), average daily gain (average 186 ± 29.4 g/day; *p* = 0.953), carcass weight (average 17.3 ± 1.64 kg; *p* = 0.261) and carcass yield (average 49.4 ± 5.3%; *p* = 0.582) were comparable between treatments.

### 3.2. Muscle Chemical Parameters and Oxidative Stability

Intramuscular fat content and muscle pH were not affected by the addition of Tara or Mimosa tannin extract to the basal diet (Table 2). The muscle concentration of γ-Tocopherol was increased by the diet containing Tara extract compared to the other treatments (0.013), while the other detected tocopherols were not affected (*p* < 0.05). Among the main FAs classes (Table 2), only the monounsaturated fatty acids (MUFA) were found at lower concentration (*p* < 0.05) in muscle from Tara group as compared to Control and Mimosa groups. A trend (*p* < 0.1) towards an increased total polyunsaturated fatty acids (PUFA) content in the muscle from Tara lambs is worth of mentioning. The main individual FAs were shown in the Supplementary Table S1. Oleic acid (C18:1 c9) was the most represented fatty acid among MUFA (82.7 ± 1.11% of total MUFA on average in the three groups) and its proportion was lower in T group compared to C lambs (*p* = 0.027). Similar to oleic acid, C16:1 c7, C16:1 c9, C17:0 ante and C17:1 c9 were found at lower proportion in T muscle compared to Control (*p* = 0.05). Moreover, the sum of all identified trans-18:1 was significantly lower in meat from T lambs as compared to the other two groups (2.25% vs. 3.13% and 3.26% FAME respectively for T, C and M groups; *p* < 0.05; data not shown). Among PUFA, linoleic acid (C18:2 c9 c12) showed a trend towards a higher proportion in meat from Tara lambs as compared to Control lambs (*p* = 0.08).

Table 3 shows the oxidative stability parameters measured in meat samples from the three dietary treatments during 7 days of aerobic refrigerated storage. The dietary supplementation with Tara or Mimosa tannins did not produce relevant effects (*p* > 0.05) on color descriptors and TBARS. Metmyoglobin percentage was the only meat stability parameter affected by dietary treatment, showing a significantly (*p* < 0.048) lower value in meat from M-fed lambs compared to C-fed lambs. Meat yellowness (b*) was not affected by the diet supplied to lambs or by the time of storage (*p* > 0.05). Meat lightness (L*), redness (a*) and hue angle (H*) values were affected by the time of storage (*p* < 0.001); regarding saturation (C*), a trend (*p* = 0.07) towards a lower value on day 7 as compared to day 0 has been observed. Metmyoglobin percentage and TBARS values were affected by the time of storage, with increasing values from day 0 to day 7.

### 3.3. Microbiological Results

**Figure 1:**
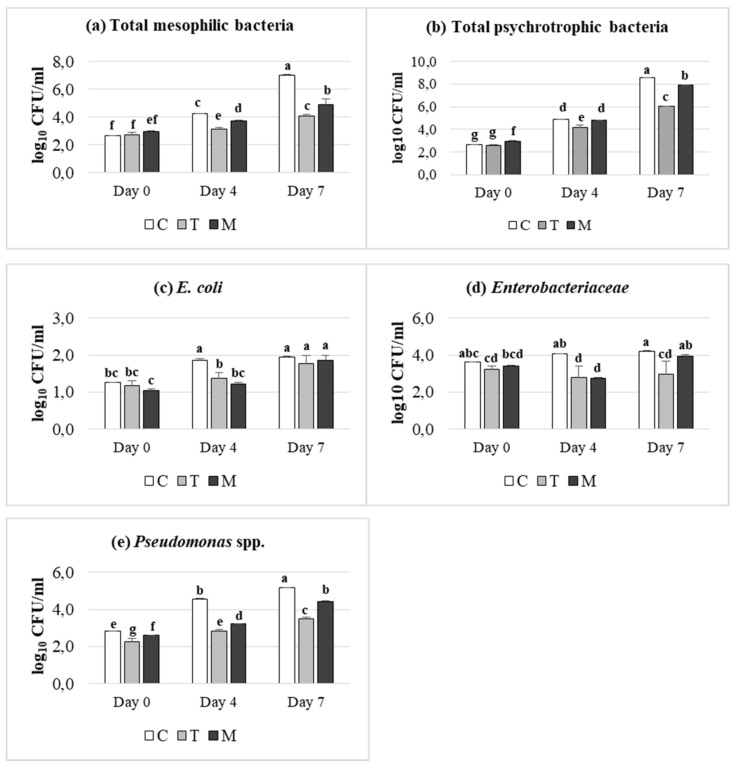
Effect of dietary treatment (Control (C), Tara (T) or Mimosa (M)) and time of storage (days 0, 4 and 7) on microbial counts expressed as log10 CFU/mL: (**a**) total mesophilic bacteria; (**b**) total psychrotrophic bacteria; (**c**) *E. coli*; (**d**) *Enterobacteriaceae*; (**e**) *Pseudomonas* spp. **a**–**g**: Values with different superscripts are significantly different (*p* ≤ 0.05).

The effects of dietary treatment (C, T, M) and of storage condition (days 0, 4 and 7) on microbial counts, expressed as log_10_ CFU/mL, are reported in Figure 1. As expected, all microbial groups analyzed, increased in meat samples during the refrigerated storage, even a dietary supplementation dependent effect was achieved. In detail, the cell density of all microbial groups was significantly lower in tannin treatments (*p* ≤ 0.05) than control one, at 4 days of storage. It is interesting to note that *Enterobacteriaceae* population showed a dramatically decrease in meat from T and M-fed lambs, which was maintained till 7 days, in contrast to control, which exhibited an increasing trend during storage. A similar trend was observed after 7 days of refrigerated storage in meat from T and M-fed lambs, despite a slightly higher cell density than day 4 was registered. Overall, T supplementation was more effective than M treatment, indicating a higher inhibiting effect against meat pathogens, especially versus *Enterobacteriaceae*.

### 3.4. Isolation and Genetic Identification of Psychrotrophic Bacteria

**Figure 2:**
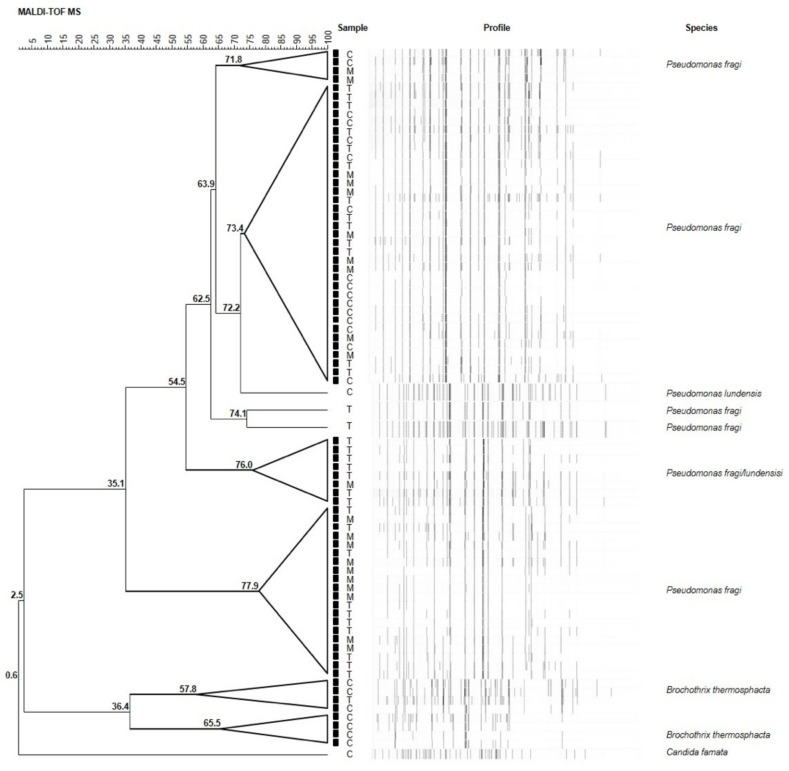
UPGMA (unweighted pair group method with arithmetic mean) dendrogram of the MALDI-TOF MS analyses of seventy-nine randomly selected meat samples. Node values indicate the average percentage of similarity based on MALDI-TOF MS profiles. The tree was made with BioNumerics version 5.1.

**Figure 3:**
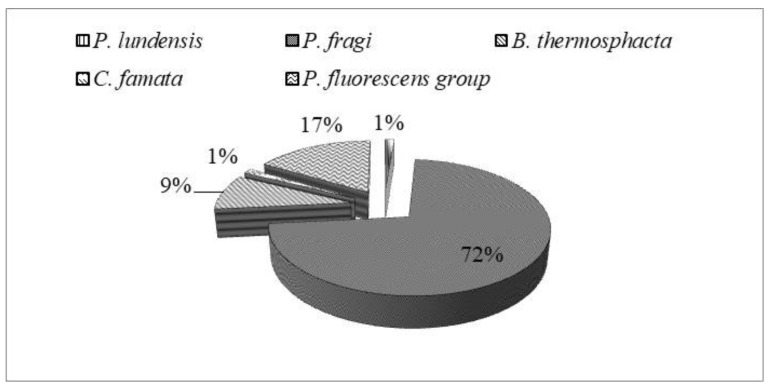
Occurrence (percentage, %) of dominant spoilage species in meat samples.

Seventy-nine randomly selected isolates were subsequently subjected to MALDI-TOF MS analysis for identification at species level. Results are illustrated in Figure 2. Overall, among isolates, 57 (72%) strains were ascribed to *P. fragi*, 7 (9%) to *Brochothrix thermosphacta*, 1 (1%) to *Pseudomonas lundensis*, and 1 (1%) to *Candida famata* (Figure 3). The remaining 13 (17%) strains were ascribed to the members of *Pseudomonas fluorescens* group, representing the most found group in T and M samples. They will be subjected to sequencing of the 16S rDNA in order to confirm their affiliation.

**Figure 4:**
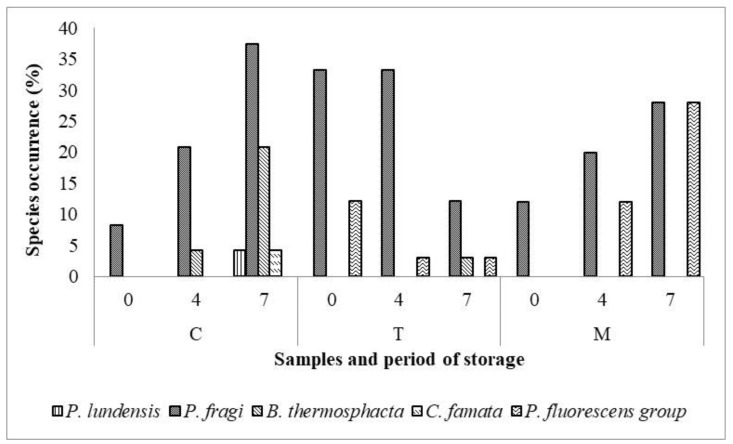
Occurrence (percentage, %) of dominant spoilage species in Tara (T), Mimosa (M) and Control (C) meat samples during the refrigerated storage.

Zooming in to the prevalence of spoilage bacteria in M, T, and C samples, as represented in Figure 4, the *P. fragi* was the most abundant species in all samples, followed by *B. thermosphacta*, *C. famata* and *P. lundensis*. Only in T samples was a significant reduction of spoilage bacteria revealed during storage. On the contrary, a qualitative and quantitative increase of spoilage bacteria was observed in C samples at 7 days of refrigerated storage; indeed, *P. lundensis* and *C. famata* species were detected only in C samples.

## 4. Discussion

### 4.1. Fatty Acid Composition

Several studies have showed that dietary tannins can favorably modulate ruminal biohydrogenation (BH) of dietary polyunsaturated fatty acids. Depending on the steps in which tannins affect the dietary PUFA biohydrogenation process, different results on rumen fatty acids outflow were observed. Literature shows an increase of PUFA [14,22] when the initial steps of the BH are inhibited. On the other side, an increase of the BH intermediates, rumenic, vaccenic and other trans-18:1 acids and a decrease of stearic acid might result from the inhibition of the last step of BH [11,22]. In the barley-based diet supplied to lambs in the present study, linoleic acid represents the main PUFA substrate for BH. The addition of Tara or Mimosa extracts to the diet produced only minor effects on dietary PUFA biohydrogenation. Indeed, no significant (*p* > 0.05) effects were observed on meat fatty acids composition for the main products of linoleic acid BH, vaccenic and rumenic acids. However, other FAs (i.e., C17:0 anteiso, C17:1 c9 and C18:1 t10), also linked to BH process, were depressed by the tannin-hydrolysable supplementation (i.e., Tara). Moreover, a significant effect was observed on the total trans-18:1 FAs, which resulted in a lower content in meat from T lambs as compared to the other two groups. In the light of these findings, it would seem that the hydrolysable tannins have influenced the rumen microbial population. Indeed, variations in C17:0 anteiso and C17:1 c9 indicate changes in microbial growth as they are synthesized by rumen microorganisms and included in their membranes [23], whereas, the effect on C18:1 t10 could be explained by a shift of rumen microorganism community, which can produce mainly C18:1 t10 at the expense of the C18:1 t11 [24]. Differently, condensed tannins from Mimosa did not affect meat fatty acids composition, as assessed by the lack of significance between meat from M and C groups.

The most relevant results on meat fatty acid composition concern the significant effect of T extract addition to lambs’ diet on meat total MUFA and oleic acid and the trend observed for linoleic acid. According to Wood et al. [25], an increase in the proportion of oleic acid and a decrease in the proportion of linoleic acid in neutral lipid as fat deposition accelerates is often observed, both in muscle and in adipose tissue. In the present study, the Pearson coefficients between intramuscular fat (IMF) content and oleic and linoleic acids were highly significant (respectively for oleic and linoleic: r = 0.752, *p* = 0.001; r = −0.797, *p* < 0.001). It may be inferred that, in our experimental conditions, the effect of the diet on oleic and linoleic acids percentages could be the result of the slightly but not significantly (*p* > 0.05; Table 2) different IMF level observed in the meat. Indeed, meat from T lambs contained, on average, about fifty percent of intramuscular fat as compared to meat from C lambs.

### 4.2. Oxidative Stability of Meat

As is well known, the meat oxidative stability depends on the balance between muscle oxidizable substrates and antioxidants defenses [26]. Vitamin E is a powerful lipophilic antioxidant, which can effectively delay the deterioration of the meat during storage [27]. In our experiment, a higher concentration of γ-Tocopherol was found in muscle from Tara treatment compared to Control and Mimosa groups. Considering that the basal diet was the same for all the treatment and the supplemented tannins did not contain tocopherols because they were produced by aqueous maceration, all lambs ingested similar concentration of vitamin E. In turn, a change of tocopherols in muscle was unexpected. However, the increase of γ-Tocopherol in T treatment did not lead to an improvement in oxidative stability. Indeed, comparable results were observed for color descriptors and lipid oxidation (TBARS values). This lack of results can be partially explained by the very low concentration of γ-Tocopherol compared to the other vitamin E isomers (i.e., α- and δ-tocopherols) and the lower antioxidant action than α-tocopherol [27].

Previous studies have reported that tannin supplementation of the ruminant diet has positive effects on the oxidative stability of meat [28,29]. However, in the present study, these desired effects of dietary tannins were not observed. These discrepancies can be due to many factors, such as the type of tannins, the dose used and the interaction with the basal diet, which do not allow for reaching clear conclusions on the effects of tannins on meat oxidative stability [18].

The metmyoglobin formation was reduced by condensed tannins supplementation (M groups) as compared to meat from lambs receiving no tannins or hydrolysable tannins (C and T groups respectively). Similarly, Luciano et al. [28] observed a reduction of metmyoglobin formation during refrigerated storage of meat from lambs fed a diet supplemented with condensed tannin extract (quebracho; *Schinopsis lorentzii*). Nevertheless, unlike the present study, these authors also reported an improvement in color descriptors of meat.

Regardless of the dietary treatments, color descriptors, TBARS values and metmyoglobin percentage changed during time of storage, which was expected and in agreement with literature [16,28,29]. However, it should be underlined that the TBARS values were always below the threshold of 2 mg MDA/kg, at which consumers can perceive off-flavors and rancidity of meat [30].

### 4.3. Microbiological Quality of the Lamb Meat

In recent years, the application of plant extracts to meat, as natural antioxidants and antimicrobial agents, has become a topic of great concern for meat industry [1,2,3]. On the other hand, few studies have investigated on the relationships between dietary consumption of plant extracts rich in secondary compounds and meat microbial spoilage. An interesting antimicrobial effect has been observed in meat when lambs received rosemary extract [31,32] or thyme [33] or in carcasses from rabbit supplemented with oregano essential oil [34]. Looking at the microbiological quality of the lamb meat, our data demonstrated a positive effect of tannin-rich feed on microbial composition of lamb meat, stored for 7 days at refrigerated condition. A significant decrease of the overall load of microbial groups investigated was observed mostly in T samples, suggesting the antimicrobial properties of hydrolysable tannins or of some product of their degradation, inside the muscular tissue. Indeed, previous studies reviewed by Smeriglio and co-workers [35], have already demonstrated the antimicrobial activity of tannin compounds against a wide range of gram-positive and gram-negative bacterial strains by complexing with proteins through both covalent and non-covalent interactions, and with polysaccharides [36]. Our data demonstrated that the occurrence of *P. fragi* species was dramatically reduced at 7 days of storage. It is well established that *P. fluorescens* group is the main cause of meat spoilage during aerobic storage condition [37]. Together with enterobacteria, these spoilers are well-known to produce several volatile organic compounds causing off-odors upon storage [38]. It is interesting to highlight that both Tara and Mimosa tannin-extracts supplemented in lamb’s diets inhibited the growth of *P. lundensis* and *C. famata* species, which were detected only in the Control groups samples. However, *B. thermosphacta*, well known as a dominant organism in meat spoilage, becoming ubiquitous throughout the meat production chain [39], was detected both the C and the T samples after 7 days of storage. Its ability to grow at refrigeration condition and its tolerance to high-salt and low-pH conditions involve production of organoleptically unpleasant compounds [40] in fresh and cured meats, and fish products [41], playing an important role in shortening the shelf-life of these products [39]. The occurrence of *C. famata* in lamb meat could be originated by spoiled carcasses representing the main responsible of pink pigments formation in many meat products [42]. Even though polyphenols in muscle were not measured in this study, it may be hypothesized that metabolites from Tara or from Mimosa tannins reached the muscular tissue operating an antibacterial activity during meat storage.

## 5. Conclusions

The inclusion of Mimosa and Tara tannins in the lamb’s diet showed mild effects on meat fatty acid profile and on oxidative stability of meat. However, the most relevant result was the antimicrobial effect of Tara extract on spoilage bacteria. To the best of our knowledge, this is the first study addressing the positive effects of tannin-extract supplied through the diet on microbial population of lamb meat during the shelf-life. The ability of tannin molecules to exert positive effects on lamb’s meat represents a promising strategy to improve the quality and the shelf-life of the final product.

## Figures and Tables

**Table 1:** Chemical composition of the basal diet.

	Basal Diet
Dry matter (DM), g/100 g as-fed	89.65
Crude protein, g/100 g DM	15.67
Ether extract, g/100 g DM	2.68
Neutral detergent fibre, g/100 g DM	30.36
Acid detergent fibre, g/100 g DM	15.97
Acid detergent lignin, g/100 g DM	3.62
Ash, g/100 g DM	7.01
Total tocopherols, μg/g DM	13.08
α-Tocopherol, % of total tocopherols	98.75
γ-Tocopherol, % of total tocopherols	1.16
δ-tocopherol, % of total tocopherols	0.08
Fatty acids, g/kg DM	
14:0	0.06
16:0	5.82
18:0	1.51
18:1 n-9	8.94
18:2 n-6	28.03
18:3 n-3	0.07
20:0	0.16

**Table 2:** Effect of the dietary treatment ^1^ on meat quality parameters.

Item ^2^	C	T	M	SEM ^3^	Diet Effect
IMF^3^, g/100 g muscle	2.19	1.15	1.87	0.135	0.136
pH	5.94	5.88	6.00	0.040	0.490
α-Tocopherol, ng/g of muscle	258	468	340	44.40	0.149
γ-Tocopherol, ng/g of muscle	1.82 ^b^	3.40 ^a^	1.83	0.278	0.013
δ-Tocopherol, ng/g of muscle	18.6	20.2	29.0	2.530	0.200
Σ Tocopherols, ng/g of muscle	278	492	371	46.00	0.167
SFA, g/100g total FAME	39.5	39.9	40.4	0.593	0.841
MUFA, g/100g total FAME	47.0 ^a^	41.4 ^b^	45.5 ^a^	0.840	0.006
PUFA, g/100g total FAME	10.3	15.6	10.9	1.120	0.093

^1^ C = concentrate-based diet; T and M mean C diet + 4% tannin extract from either Tara or Mimosa. ^2^ IMF = intramuscular fat; SFA = saturated fatty acids; MUFA = monounsaturated fatty acids; PUFA = polyunsaturated fatty acids; FAME = fatty acid methyl esters. ^3^ SEM = standard error of mean. ^a,b^ Within a row, different superscript letters indicate differences (*p* ≤ 0.05) between dietary treatments tested using the Tukey’s adjustment for multiple comparisons.

**Table 3:** Effect of the dietary treatment and time of storage on the oxidative stability parameters of meat.

	Dietary Treatment (D) ^1^			Time of Storage (T) ^2^			SEM ^3^	*p* Values ^4^		
	C	T	M	0	4	7		D	T	D × T
L* values	42.5	42.9	41.7	40.8 ^b^	42.8 ^ab^	43.5 ^a^	0.455	0.750	<0.001	0.029
a* values	12.5	12.7	12.2	13.9 ^a^	12.2 ^b^	11.3 ^b^	0.279	0.786	<0.001	0.131
b* values	11.8	11.6	10.8	11.1	11.5	11.6	0.223	0.396	0.479	0.084
C* values	17.3	17.2	16.4	17.8	16.8	16.2	0.314	0.579	0.035 ^6^	0.085
H* values	43.6	42.4	41.4	38.3 ^c^	43.2 ^b^	46.0 ^a^	0.602	0.225	<0.001	0.945
MetMb, % of Mb	48.4 ^a^	46.0 ^ab^	43.8 ^b^	38.3 ^c^	48.3 ^b^	51.6 ^a^	1.02	0.048	<0.001	0.499
TBARS ^5^, mg/kg	0.76	0.83	0.71	0.18 ^c^	0.64 ^b^	1.48 ^a^	0.106	0.857	<0.001	0.884

^1^ C = concentrate-based diet; T and M mean C diet + 4% tannin extract from either Tara or Mimosa. ^2^ Time 0, 4, 7 = days of storage at 4 °C under aerobic conditions (raw meat slices) ^3^ SEM = standard error of mean. ^4^ *p* values for the effects of the dietary treatment (D), time of storage (T) and of the D × T interaction. ^5^ Lipid oxidation, measured as TBARS values. ^6^ No significant differences were found for multiple comparisons using Tukey’s method. ^a–c^ Within row, different superscript letters indicate differences (*p* < 0.05) between dietary treatments or times of storage tested using the Tukey’s adjustment for multiple comparisons.

## Supplementary Materials

The following are available online at https://www.mdpi.com/2304-8158/8/10/469/s1, Table S1: Effect of the dietary treatment^1^ on the main fatty acids (g/100 of total FAME) of meat.
