# Supplementary material for: Dietary Supplementation of Tannin-Extracts to Lambs: Effects on Meat Fatty Acids Composition and Stability and on Microbial Characteristics
Source: Foods. 2019 Oct 10;8(10):469. doi: 10.3390/foods8100469 (PMC6836261; doi:10.3390/foods8100469)
Supplement: Supplementary file 1 [file foods-08-00469-s001.pdf]

Dietary Supplementation of Tannin-Extracts to Lambs: Effects on Meat Fatty Acids Composition and Stability and on Microbial Characteristics

Luisa Biondi [1], Cinzia L. Randazzo [1,*], Nunziatina Russo [1], Alessandra Pino [1], Antonio Natalello [1], Koenraad Van Hoorde [2,3] and Cinzia Caggia [1]

[1] Department of Agriculture, Food and Environment, University of Catania, Catania 95123, Italy; lubiondi@unict.it (L.B.); nunziatinarusso83@gmail.com (N.R.); alessandra.pino@unict.it (A.P.); antonio.natalello@unict.it (A.N.); ccaggia@unict.it (C.C.)

[2] Service Foodborne Pathogens, Sciensano, Brussels B-1050, Belgium

[3] Faculty of Bioscience Engineering, Department of Biotechnology, Laboratory of Brewing Science and Technology, Ghent University, Ghent B-9000, Belgium, Koenraad.VanHoorde@sciensano.be

Table S1. Effect of the dietary treatment[1] on the main fatty acids (g/100 of total FAME) of meat.

	Dietary Treatment			SEM [2]	*p* Values
	C	T	M		
C12:0	**0.13**	0.12	0.13	0.005	0.658
C14:0	3.02	2.74	2.78	0.109	0.547
C16:0	24.03	23.76	23.51	0.437	0.905
C16:0 *iso*	0.12	0.11	0.11	0.003	0.391
C16:1 c7	0.30 [a]	0.24 [b]	0.27 [ab]	0.009	0.018
C16:1 c9	1.90 [a]	1.52 [b]	1.62 [ab]	0.068	0.050
C17:0	1.14	0.93	1.05	0.056	0.333
C17:0 *iso*	0.35	0.34	0.35	0.008	0.819
C17:0 *anteiso*	0.47 [a]	0.39 [b]	0.45 [ab]	0.011	0.013
C17:1 c9	0.71 [a]	0.51 [b]	0.59 [ab]	0.036	0.051
C18:0	12.05	13.05	13.67	0.298	0.069
C18:1 c9	38.54 [a]	34.47 [b]	37.72 [ab]	0.699	0.027
C18:1 c11	1.53	1.62	1.34	0.064	0.181
C18:1 c12	0.43	0.36	0.32	0.022	0.119
C18:1 t9	0.26	0.23	0.31	0.016	0.137
C18:1 t10	1.67 [ab]	1.09 [b]	1.91 [a]	0.137	0.026
C18:1 t11	0.77	0.57	0.59	0.052	0.212
C18:2 c9 t11	0.44	0.31	0.32	0.027	0.071
C18:2 c9 c12	6.78	9.85	7.34	0.609	0.080
C18:3 n-3	0.52	0.57	0.54	0.018	0.580
C20:4 n-6	1.30	2.80	1.33	0.347	0.125
C22:5 n-3	0.20	0.46	0.20	0.065	0.160

[1] C = concentrate-based diet; T and M mean C diet + 4% tannin extract from either Tara or Mimosa. [2] SEM = standard error of mean. [a, b] Within a row, different superscript letters indicate differences ($p \leqslant 0.05$) between dietary treatments tested using the Tukey's adjustment for multiple comparisons.
